# Individual and contextual correlates of mosquito net use among women in Nigeria

**DOI:** 10.1186/s12936-020-03219-3

**Published:** 2020-04-07

**Authors:** Sulaimon T. Adedokun, Olalekan A. Uthman

**Affiliations:** 1 Department of Demography and Social Statistics, Obafemi Awolowo University, Ile-Ife, Nigeria; 2 Warwick-Centre for Applied Health Research and Delivery (WCAHRD), Division of Health Sciences, University of Warwick Medical School, Coventry, UK; 3 Centre for Evidence-based Health Care, Faculty of Medicine and Health Sciences, Stellenbosch University, Cape Town, South Africa

**Keywords:** Nets, Mosquito, Use, Malaria, Contextual, Multilevel, Utilization, Model

## Abstract

**Background:**

Malaria has been described as an urgent public health priority with almost half of the world’s population being at risk. Use of insecticide-treated nets is considered one of the effective ways of preventing malaria. Nigeria, which is ranked among the five countries that are responsible for almost half of the global malaria cases, has less than half of its women population using mosquito nets. This study examined the effects of individual and contextual factors on the use of mosquito nets among women of reproductive age in Nigeria.

**Methods:**

This study used data obtained from 2015 Nigeria Malaria Indicator Survey (NMIS) which involved 6048 women aged 15–49 who possessed at least one mosquito net. Multilevel binary logistic regression models were applied in the multivariable analysis.

**Results:**

About 53% of the women used mosquito nets with more than 60% of uneducated and poor women in this category. The use of mosquito nets was significantly associated with being from poor households, having knowledge about the cause of malaria, having access to malaria messages, possessing knowledge about the efficacy of malaria prevention drugs during pregnancy, having knowledge about the importance of tests to detect malaria, maintaining small household size and living in the most socioeconomically disadvantaged communities and states.

**Conclusions:**

The study revealed that mosquito net use among women in Nigeria is affected by individual and contextual factors. It is important for policy makers to design a mosquito-net-use model which would take individual and contextual factors into consideration.

## Background

Malaria, which is linked to high morbidity and mortality particularly among women and children, has been described as an urgent public health priority [1]. In 2017, there were 219 million cases of malaria which resulted in 435,000 deaths [2]. It has been reported that almost half of the world’s population was at risk as at 2017 [2]. Malaria among women of reproductive age has attracted attention due to its effects on the women and their reproductive process. In this light, malaria infection during pregnancy has been described as a major public health challenge with significant implications for pregnant women, the fetus and the new born [3].

As a result of malaria, an estimated 10,000 women and 200,000 infants die annually in Africa [4]. In order to prevent and control malaria, huge resources have been committed to different initiatives and strategies by international bodies. In 2017, about 3.1 billion dollars was spent on such programmes with 28% provided by governments of endemic countries [2]. One of these strategies is the Global Technical Strategy for Malaria 2016–2030 of the World Health Organization (WHO). It was adopted in 2015 by the World Health Assembly and designed to achieve the following by 2030: (i) reduce malaria mortality rates by at least 90% (ii) eliminate malaria from at least 35 countries and (iii) prevent resurgence of malaria in all countries that are malaria-free [5]. Guided by this Global Technical Strategy, many agencies including the WHO, Global Fund, Roll Back Malaria support countries to control and eliminate malaria [6].

Malaria has been one of the major health concerns in Nigeria. In 2017, the country was ranked among the five countries that were responsible for almost half of the global malaria cases [2]. It has also witnessed the establishment of programmes directed at preventing and controlling malaria. One of these is National Malaria Strategic Plan 2014–2020. The programme, among other things, aimed at ensuring that: at least 80% of targeted populations utilize appropriate preventive measures by 2020; all persons with malaria receive prompt treatment with an effective anti-malarial; and at least 80% of the population practice appropriate malaria prevention and management by 2020 [7]. The preventive aspect of the programme included the promotion of the use of insecticide-treated mosquito nets [8]. Since 2011, about 24 million nets have been provided free of charge in the country [9]. Despite these efforts at promoting net use and the resources committed to the programme, less than half of the women in the country use mosquito nets [10].

Attempts have been made by researchers to examine the factors influencing mosquito net use among women or caregivers that possessed mosquito nets. Some of the factors attributed to this include age, education, household wealth [11–13], household size, distance to health facility, ideational factors [10, 14, 15], knowledge about efficacy of mosquito nets to prevent malaria, source of knowledge of mosquito nets and socioeconomic class [16, 17]. Only few of these studies considered the effects of contextual factors on mosquito net use. Since individuals are nested within the community and the community is nested within the state, considering individual factors alone would not provide adequate explanation for mosquito nets use. This study aimed at filling this gap by examining the effects of individual and contextual factors on mosquito net use among women of reproductive age in Nigeria.

## Methods

### Study design

The data set used in this study was obtained from 2015 Nigeria Malaria Indicator Survey (NMIS) which is cross-sectional and provides information on malaria prevalence and indicators at regional and state levels in the country.

### Sampling technique

A two-stage sampling method was used to select the sample for the 2015 NMIS. Based on information from the last census exercise in the country, each state was divided into local government areas (LGAs) and each local government area was divided into localities. From each locality, enumeration areas (EAs) also referred to as clusters were carved out. The first stage involved random selection of 9 clusters from each state including the Federal Capital Territory (FCT). Altogether, 333 clusters were selected resulting into 138 in urban areas and 195 in rural areas. In the second stage, 25 households were selected from each cluster using systematic sampling technique. Eligible respondents were thereafter selected for interview in these households. Details of sampling method used have been published elsewhere [18].

### Data collection

Information from women between ages 15 and 49 years who were either permanent residents of the households or visitors present in the households in the night preceding the survey was obtained through administration of questionnaire. For adequate information gathering, the questionnaire was translated into three major languages in the country namely Yoruba, Igbo and Hausa. Information sought through the questionnaire was on background characteristics, birth history and child mortality, malaria prevention and treatment, antenatal care, malaria prevention for most recent birth and pregnancy and knowledge about malaria, among others. Details of data collection method have been published elsewhere [18].

### Outcome variable

This study was limited to women between ages 15 and 49 years who possessed at least one mosquito net. Women who reported sleeping under a treated mosquito net in the night preceding the survey were categorized as using mosquito nets while those who did not sleep under a treated mosquito net were categorized as not using mosquito nets. The former was thereafter defined as a binary variable with a value of 1 and the latter was coded 0.

### Explanatory variables

#### Individual-level factors

At the individual level, the following variables were considered: age, education, household wealth, knowledge about causes of malaria, exposure to malaria messages, knowledge about the efficacy of mosquito nets, knowledge about the efficacy of malaria prevention drugs, knowledge about the importance of tests to detect malaria, knowledge about the efficacy of artemisinin-based combination therapy (ACT) and number of household members. Age was categorized into 15–24, 25–34 and 35+ years. Education was defined as no education, primary and secondary or higher. Household wealth was primarily defined as wealth index in the DHS data set with five categories. Wealth index was obtained from the measurements of household items possessed by each household. Such items include radio, television, car, toilet facilities, type of floor/roofing, water source. In order to ensure easy interpretations of results, principal component analysis was applied and the index reclassified into three (poor, middle and rich). Knowledge about causes of malaria was defined as ‘Yes’ for women who reported that mosquitoes cause malaria and ‘No’ for those who reported otherwise. Exposure to malaria messages defined as ‘Yes’ for those who heard or saw messages about malaria 6 months preceding the survey and ‘No’ for those who were not exposed to such messages. Knowledge about the efficacy of mosquito nets was measured as ‘Yes’ for women who agreed that the chances of getting malaria were the same whether mosquito nets were used or not and ‘No’ for those who either disagreed or claimed that they did not know. Knowledge about the efficacy of malaria prevention drugs was defined as ‘Yes’ for those who agreed that drugs used by pregnant women to prevent malaria were effective and ‘No’ for those who disagreed or reported that they did not know. Knowledge about the importance of tests to detect malaria was defined as ‘Yes’ for women who agreed that tests are a good way to detect malaria and ‘No’ for those who disagreed or claimed that they did not know. With respect to the efficacy of ACT, women who agreed that it works quickly were defined as ‘Yes’ while those who disagreed or reported they did not know were defined as ‘No’. Number of household members was categorized into less than 5 and 5 or more.

#### Community-level factors

The variables considered at community level include residence, region and socioeconomic status. Residence was categorized into urban and rural. Region was grouped into North Central, Northeast, Northwest, Southeast, Southwest, South-South and Southwest. Socioeconomic status was obtained by applying principal component analysis technique to measure the proportions of individuals who were uneducated, poor and unemployed within the communities. The result was thereafter categorized into tertile 1 (least disadvantaged), tertile 2 and tertile 3 (most disadvantaged).

#### State-level factors

State-level factors comprise socioeconomic status at state level which was obtained by applying principal component analysis technique to measure the proportions of individuals who were uneducated, poor and unemployed within the states. The result was thereafter categorized into tertile 1 (least disadvantaged), tertile 2 and tertile 3 (most disadvantaged).

### Statistical analysis

#### Descriptive statistics

Results from the descriptive analysis carried out comprise percentage distribution of all the explanatory variables against the outcome variable. Chi-Square test was used to obtain p-values which reflect the level of significance of each variable.

#### Modelling approaches

A multilevel binary logistic regression model which considered factors at individual, community and state levels was applied in the analysis. The hierarchical nature of the data informed the adoption of multi-level modelling. Four models were thereafter generated. The first model, which is referred to as empty model, consists of no explanatory variables and was specified with the aim of decomposing the amount of variance that occurred between community and state levels. The second model comprised individual-level variables while the third model comprised both individual and community-level variables. The fourth model which is referred to as the full model consisted of all the explanatory variables at the three levels. While the fixed effects were measured by odds ratios (OR) including their corresponding 95% credible intervals, random effects were measured by three parameters: intra-cluster correlation coefficient (ICC), variance partition coefficient (VPC) and median odds ratio (MOR). MOR is used to measure the unexplained cluster heterogeneity. Details of the methods used to obtain MOR have been published elsewhere [19, 20].

#### Model fit and specification

Some statistical operations were performed to ensure that the models were in order and adequately specified. While Bayesian Deviance Information Criterion (DIC) was used to test the goodness of fit of the model, variance inflation factor (VIF) was used to check multicollinearity. All the multilevel modelling operations were conducted through MLwiN 2.35 [21] using Stata statistical software [22]. In addition, Markov Chain Monte Carlo (MCMC) estimation was included in the operation [23].

## Results

### Descriptive analysis

In Table 1, a descriptive analysis of the women’s characteristics is presented. The study involved 6048 women between ages 15 and 49 years defined as level 1, nested within 896 communities (level 2) and from 37 states (level 3) in Nigeria. About 53% of the women used mosquito nets and most of them (54%) are between 25 and 34 years of age. More than 60% of uneducated and poor women used mosquito nets. Majority of those who hold the opinion that malaria is caused by mosquitoes (59%) including those that have heard or seen messages about malaria (60%) made use of mosquito nets. Most of those who claim that the chances of getting malaria is the same whether mosquito nets are used or not (61%) made use of mosquito nets. Close to 60% of women who claimed that drugs used by pregnant women to prevent malaria are effective use mosquito nets. Also, those who support the notion that tests are a good way to determine malaria status of individuals use mosquito nets. About 60% of those who affirm that ACT works quickly in the treatment of malaria use mosquito nets. Most women from rural area (58%) and regions such as North Central (53%), Northeast (64%) and Northwest (62%) use mosquito nets. Preponderance of women from most economically disadvantaged communities (64%) and states (67%) use mosquito nets.

**Table 1:** Use of mosquito nets among women at different levels of independent variables

Variable	Used mosquito nets			
	No	Yes	Total	p-value
	N (%)	N (%)	N (%)	
Individual-level factors	2860 (47.3)	3188 (52.7)	6048 (100.0)	
Age				
15–24	1061 (47.8)	1161 (52.2)	2222 (100.0)	
25–34	1027 (46.5)	1180 (53.5)	2207 (100.0)	
35+	772 (47.7)	847 (52.3)	1619 (100.0)	0.672
Education				
No education	944 (39.5)	1446 (60.5)	2390 (100.0)	
Primary	423 (43.7)	546 (56.3)	969 (100.0)	
Secondary/higher	1493 (55.5)	1196 (44.5)	2689 (100.0)	< 0.001
Household wealth index				
Poor	731 (36.2)	1286 (63.8)	2017 (100.0)	
Middle	881 (43.7)	1134 (56.3)	2015 (100.0)	
Rich	1248 (61.9)	768 (38.1)	2016 (100.0)	< 0.001
Mosquito causes malaria				
No	948 (70.6)	394 (29.4)	1342 (100.0)	
Yes	1912 (40.6)	2794 (59.4)	4706 (100.0)	< 0.001
Exposed to malaria messages				
No	1951 (51.4)	1846 (48.6)	3797 (100.0)	
Yes	909 (40.4)	1342 (59.6)	2251 (100.0)	< 0.001
Chances of getting malaria are the same				
No	1745 (55.2)	1414 (44.8)	3159 (100.0)	
Yes	1115 (38.6)	1774 (61.4)	2889 (100.0)	< 0.001
Drugs for preventing malaria in pregnancy are effective				
No	892 (74.6)	304 (25.4)	1196 (100.0)	
Yes	1968 (40.6)	2884 (59.4)	4852 (100.0)	< 0.001
Tests are a good way to detect malaria				
No	823 (75.8)	263 (24.2)	1086 (100.0)	
Yes	2037 (41.1)	2925 (58.9)	4962 (100.0)	< 0.001
ACT is effective in treating malaria				
No	1250 (60.7)	811 (39.3)	2061 (100.0)	
Yes	1610 (40.4)	2377 (59.6)	3987 (100.0)	< 0.001
Number of household members				
< 5	817 (48.6)	864 (51.4)	1681 (100.0)	
5+	2043 (46.8)	2324 (53.2)	4367 (100.0)	0.204
Community-level factors				
Residence				
Urban	1276 (55.1)	1041 (44.9)	2317 (100.0)	
Rural	1584 (42.5)	2147 (57.5)	3731 (100.0)	< 0.001
Region				
North Central	454 (47.1)	509 (52.9)	963 (100.0)	
Northeast	446 (36.4)	781 (63.6)	1227 (100.0)	
Northwest	636 (38.4)	1.019 (61.6)	1655 (100.0)	
Southeast	434 (64.9)	235 (35.1)	669 (100.0)	
South-South	433 (54.0)	369 (46.0)	802 (100.0)	
Southwest	457 (62.4)	275 (37.6)	732 (100.0)	< 0.001
Socioeconomic disadvantage				
Tertile 1 (least disadvantaged)	1295 (63.3)	752 (36.7)	2047 (100.0)	
Tertile 2	847 (42.3)	1156 (57.7)	2003 (100.0)	
Tertile 3 (most disadvantaged)	718 (35.9)	1280 (64.1)	1998 (100.0)	< 0.001
State-level factors				
Socioeconomic disadvantage				
Tertile 1 (least disadvantaged)	1309 (62.8)	774 (37.2)	2083 (100.0)	
Tertile 2	903 (44.9)	1110 (55.1)	2013 (100.0)	
Tertile 3 (most disadvantaged)	648 (33.2)	1304 (66.8)	1952 (100.0)	< 0.001

#### Measures of association (fixed effects)

Table 2 shows results from the different models specified in the study. In model 4, which was specified to comprise all the independent variables, household wealth, knowledge about causes of malaria, exposure to malaria messages, knowledge about the efficacy of mosquito nets, knowledge about the efficacy of malaria prevention drugs, knowledge about the importance of tests to detect malaria, number of household members, region and socioeconomic status at community and state levels are significantly associated with use of mosquito nets. Women from poor households were 133% more likely to use mosquito nets compared with those from rich households. Women who held the opinion that mosquitoes cause malaria and those who claimed that they were exposed to malaria messages were (108% and 39%, respectively) more likely to use mosquito nets. Women who supported the view that the chances of getting malaria are the same irrespective of mosquito nets have higher likelihood of using mosquito nets. The odds of using mosquito nets increased by 138% for women who affirmed that drugs used by pregnant women to prevent malaria are effective. Women who supported the notion that tests are a good way to detect malaria were 229% more likely to use mosquito nets compared to those who have contrary opinion. The chances of using mosquito nets increased for women whose household members were less than 5 (aOR = 1.21, CrI = 1.05–1.39) and lived in the most socioeconomically disadvantaged communities (aOR = 2.41, CrI = 1.35–4.49) and states (aOR = 3.80, CrI = 1.37–9.05).

**Table 2:** Multilevel logistic regression models of correlates of mosquito net use among women in Nigeria

Variable	Model 1^a^	Model 2^b^	Model 3^c^	Model 4^d^
	aOR (CrI)	aOR (CrI)	aOR (CrI)	aOR (CrI)
Individual-level factors				
Age				
15–24		0.97 (0.82–1.14)	0.94 (0.79–1.11)	0.93 (0.78–1.09)
25–34		1.07 (0.92–1.23)	1.07 (0.91–1.26)	1.05 (0.89–1.23)
35+		1 (reference)	1 (reference)	1 (reference)
Education				
No education		1.01 (0.82–1.24)	0.87 (0.69–1.08)	0.86 (0.69–1.06)
Primary		1.23 (1.01–1.48)	1.17 (0.95–1.41)	1.16 (0.94–1.41)
Secondary/higher		1 (reference)	1 (reference)	1 (reference)
Household wealth index				
Poor		3.00 (2.33–3.87)	2.40 (1.81–3.19)	2.33 (1.76–3.05)
Middle		2.32 (1.92–2.77)	2.09 (1.71–2.56)	2.09 (1.69–2.56)
Rich		1 (reference)	1 (reference)	1 (reference)
Mosquito causes malaria				
No		1 (reference)	1 (reference)	1 (reference)
Yes		2.10 (1.71–2.53)	2.09 (1.73–2.51)	2.08 (1.71–2.58)
Exposed to malaria messages				
No		1 (reference)	1 (reference)	1 (reference)
Yes		1.39 (1.22–1.59)	1.40 (1.22–1.61)	1.39 (1.21–1.59)
Chances of getting malaria are the same				
No		1 (reference)	1 (reference)	1 (reference)
Yes		1.46 (1.25–1.67)	1.43 (1.25–1.65)	1.46 (1.25–1.69)
Drugs for preventing malaria in pregnancy are effective				
No		1 (reference)	1 (reference)	1 (reference)
Yes		2.39 (1.89–2.99)	2.43 (1.93–3.06)	2.38 (1.91–3.04)
Tests are a good way to detect malaria				
No		1 (reference)	1 (reference)	1 (reference)
Yes		3.17 (2.29–4.19)	3.23 (2.44–4.23)	3.29 (2.54–4.33)
ACT is effective in treating malaria				
No		1 (reference)	1 (reference)	1 (reference)
Yes		1.04 (0.87–1.25)	1.05 (0.91–1.23)	1.05 (0.87–1.22)
Number of household members				
< 5		1.18 (1.02–1.36)	1.21 (1.05–1.38)	1.21 (1.05–1.39)
5+		1 (reference)	1 (reference)	1 (reference)
Community-level factors				
Residence				
Urban			1 (reference)	1 (reference)
Rural			1.001 (0.72–1.29)	1.05 (0.77–1.39)
Region				
North Central			1 (reference)	1 (reference)
North East			1.29 (0.62–3.79)	1.05 (0.55–2.15)
North West			1.44 (0.61–4.03)	1.02 (0.43–2.40)
South East			0.46 (0.18–1.21)	0.76 (0.39–1.39)
South South			1.02 (0.42–2.17)	2.10 (1.05–3.84)
South West			0.76 (0.33–1.58)	1.40 (0.77–2.46)
Socioeconomic disadvantage				
Tertile 1 (least disadvantaged)			1 (reference)	1 (reference)
Tertile 2			2.07 (1.51–2.76)	1.95 (1.39–2.59)
Tertile 3 (most disadvantaged)			2.73 (1.70–4.14)	2.41 (1.35–4.49)
State-level factors				
Socioeconomic disadvantage				
Tertile 1 (least disadvantaged)				1 (reference)
Tertile 2				2.41 (1.33–4.01)
Tertile 3 (most disadvantaged)				3.80 (1.37–9.05)
Measures of variation				
State level				
Variance (SE)	0.582 (0.322–0.989)	0.551 (0.289–0.989)	0.280 (0.125–0.523)	0.192 (0.055–0.391)
Explained variation (%)	Reference	5.28	51.8	67.0
ICC (%)	13.18	12.42	6.75	4.72
MOR	2.07	2.03	1.66	1.52
Community level				
Variance (SE)	0.539 (0.409–0.689)	0.596 (0.434–0.782)	0.581 (0.432–0.762)	0.581 (0.430–0.768)
Explained variation (%)	Reference	−10.5	−7.73	−7.69
ICC (%)	25.40	25.84	20.74	19.01
MOR	2.01	2.09	2.07	2.07
Model fit statistics				
Bayesian DIC	7424	6525	6510	6514

*SE* standard error, *DIC* deviation information criterion, *CrI* credible interval, *ICC* intra-cluster correlation, *MOR* median odds ratio

^a^Model 1 is the empty model with no independent variables

^b^Model 2 is adjusted for age, education, household wealth index, knowledge about causes of malaria, exposure to malaria messages, knowledge about efficacy of mosquito nets, knowledge about efficacy of malaria prevention drugs, knowledge about importance of test to detect malaria, knowledge about efficacy of ACT and number of household members

^c^Model 3 is additionally adjusted for residence, region and community socioeconomic factors

^d^Model 4 is additionally adjusted for state socioeconomic factors

#### Measures of variation (random effects)

Table 2 also presents results of random effects. Values from the unconditional model (model 1) show that there was a significant variation in the odds of using mosquito nets across communities ($\sigma^{2} =$ 0.54, 95% CrI 0.41 to 0.69) and states ($\sigma^{2} =$ 0.58, 95% CrI 0.32 to 0.99). The intra-community and intra-state correlation coefficients indicate that 25.4% and 13.2% of the variance in odds of using mosquito nets are linked to community and state-level factors. MOR in model 4 reveals the significant effects of community and state-level factors on mosquito net use. The results show that if a woman moved to another community or state with a higher probability of mosquito net use, the likelihood of using mosquito nets would increase by 2.07 and 1.52 times respectively.

## Discussion

This study has demonstrated that the use of mosquito nets among women of reproductive age in Nigeria is influenced by both individual and contextual factors. It is revealed that women who are from poor households used mosquito nets more than their counterparts who are from rich households [24, 25]. This may be attributed to the fact that women from rich households may have devised other means of protecting themselves from mosquito bites. Such means may include living in houses fortified by mosquito-net-doors and windows and application of mosquito repellent formulae or insecticides which keep mosquitoes away from home. At the same time, such women may have the advantage of living in an environment that does not enhance mosquito breeding, unlike those from poor households who live in squalid environment with poor drainage and waste disposal systems. It is noteworthy to state that indoor residual spraying practice is relatively new in Nigeria and not all households have benefitted from it [18]. Based on these facts, poor women would avail themselves of the opportunity offered by the availability of mosquito nets.

It is also shown in the study that women who claimed that malaria is caused by mosquitoes are more likely to use mosquito nets than those who responded otherwise [26]. This indicates that knowledge about the cause of malaria is an important predictor of mosquito net use. In fact, not attributing malaria to mosquitoes may not only lead to non-use of mosquito nets, but may also lead to application of wrong therapy to treat malaria. This has serious health implications particularly when such women are pregnant. Exposure to messages about malaria impacts positively on mosquito net use as women who reported that they heard or saw messages about malaria have higher likelihood of using mosquito nets compared to women who did not have exposure to such messages [27, 28]. Messages about malaria particularly those that comprise information on means of preventing malaria, methods of treating malaria and time and cost implications of malaria generate a good platform for awareness creation. Women who have the opportunity of obtaining such information would appreciate any initiative aimed at preventing malaria.

Results further show that women who posited that the chances of getting malaria are the same whether mosquito nets are used or not were more likely to use mosquito nets. The reason for this may be that the women find other advantages of using nets such as prevention of bed bugs and other insects. Having knowledge of the efficacy of malaria prevention drugs enhances the use of mosquito nets as women who affirmed that drugs used by pregnant women to prevent malaria are effective used mosquito nets more than their counterparts who did not affirm the efficacy of such drugs. The former may have had the experience of not getting malaria during pregnancy after using the drugs. This provides them the assurance that mosquito nets would be another effective means of preventing malaria. Women who had malaria during pregnancy because they did not use malaria prevention drugs but knew about their counterparts who had the opportunity of having malaria-free experience during pregnancy due to their use of such drugs may also be among those that affirmed the efficacy of malaria prevention drugs.

Findings from the study also reveal that the likelihood of using mosquito nets is higher among women who supported the view that tests are a good way to detect malaria status of individuals. This is very important because conducting tests would not only result in correct diagnosis but would also lead to the application of appropriate therapy to treat malaria. Whenever a person attends a health service e.g. to receive an RDT test—it is an opportunity to reinforce health behaviours like net use—this is why it is good to use every opportunity of an encounter with the health service to reinforce positive health behaviours—as sustaining these behaviours is critical for malaria control. The number of household members is another factor influencing use of mosquito nets [29, 30]. Women from households with less than 5 members were more likely to use mosquito nets than those whose household members were 5 or more. This shows that the smaller the household, the higher the probability of using mosquito nets. This may be attributed to the fact that mosquito nets could only accommodate a sizeable number of household members at a time. Socioeconomic status of communities and states also influenced mosquito net use.

The odds of using mosquito nets increased tremendously for women who lived in the most socioeconomically disadvantaged communities and states. Living conditions in such communities and states are grossly poor. Lack of infrastructural facilities gives room for poor environmental conditions which enhance the growth and spread of mosquitoes. More so, the poor socioeconomic status of women in such communities and states most often obstructs their access to adequate medical care in the event of getting malaria. It could be that these women embraced the use of mosquito nets in order to avoid being caught up in such a situation.

### Policy implications

Reducing mortality rate due to malaria among women has become a global objective. Achieving this objective through the distribution of mosquito nets may be unrealistic with only half of the women who possess mosquito nets actually using them. It further generates much concern when it is considered that huge resources have been committed to the procurement of such nets by donor agencies and international organizations. Since this study focused on women who possess mosquito nets, the questions that come to mind are: why were the nets not used for the purpose for which they have been distributed? What can be done to improve mosquito net use among such women? Although the study has provided answers to the first question by revealing that individual, community and state-level factors influenced mosquito net use, answers to the second question would constitute policy recommendations which would also address components of the first question. First, there is a need to look at the way mosquito nets are being distributed. The nets should be given to those who are really in need of them particularly those who live in mosquito-infested areas and do not have means of protecting themselves against mosquitoes. A situation where the nets would be distributed to inhabitants of rich households who may already have means of protecting themselves should be avoided. This can be achieved by embarking on a pre-distribution survey which is aimed at identifying households that fulfil the condition of those that are really in need of the nets. A follow-up visitation should be carried out at regular intervals after distribution to monitor utilization among beneficiaries. Health care workers need to work in collaboration with government media houses to intensify efforts at promoting malaria prevention messages on radio and television. Such messages should also be disseminated at gatherings organized at community level. Emphasis should be placed on educating women on causes of malaria, the efficacy of drugs recommended for preventing malaria during pregnancy and the importance of going for tests to detect malaria. At the same time, attention should be paid to gender power relationships. In most cases, it is the male partner who influences a women’s behaviour about spending money, going for and using health services. This influence of men on women may need to be addressed in respect of mosquito net use. To address the challenge of non-use of mosquito nets by large households, efforts should be made to increase the number of nets per household. Government at state and federal levels should commit more resources to improving socioeconomic status of every individual on the one hand and that of states and communities on the other hand. Programmes that would alleviate poverty and improve the economic conditions of individuals should be given utmost priority. This may come in form of offering economic opportunities and providing enabling environment for small and medium-scale enterprises. Future studies on mosquito net use should consider complementing quantitative surveys with some qualitative studies in order to understand the issue more in depth.

## Study strengths and weaknesses

It should be noted that this study used a cross-sectional data set which prevented us from providing information on immediate causes of non-use of mosquito nets despite the fact that the women possessed them. Such information would be better obtained with the use of longitudinal data set which will ensure a follow-up of respondents at regular intervals. There was also the possibility of recall error during the interview with respondents as they were asked some questions that relate to events that occurred in the past. In spite of these limitations, this study has used a large data set which provided an opportunity to generalize the findings and relate such to countries in similar circumstances. The study also provided information on the role of contextual factors in mosquito net use among women.

## Conclusions

This study has revealed that the use of mosquito nets among women in Nigeria is influenced by both individual and contextual factors. There is a need to design a mosquito net utilization model that would incorporate individual and contextual components.

## Data Availability

The data set used in this study was obtained from the archive of Demographic and Health Surveys (DHS) Program which granted permission for the use of the data. The data set was ethically cleared by Nigeria Health Research Ethics Committee of the Federal Ministry of Health (NHREC). The data set is available at https://dhsprogram.com/data/dataset/Nigeria_MIS_2015.cfm?flag=0.

## Abbreviations

ACT: Artemisinin-based combination therapy
aOR: Adjusted odds ratio
CrI: Credible interval
DHS: Demographic and Health Surveys
DIC: Deviation information criterion
ESDA: Exploratory spatial data analysis
ICC: Intra-cluster correlation
MOR: Median odds ratio
NMIS: Nigeria Malaria Indicator Survey
SE: Standard error
